# A prospective descriptive cohort study of women aged ≥40 years conceiving through IVF at a tertiary care center

**DOI:** 10.3389/frph.2026.1860603

**Published:** 2026-06-24

**Authors:** Marta Domínguez-Moreno, Blanca Walsh, José De-Martín-Hernández, Ángel Chimenea, Guillermo Antiñolo, Lutgardo García-Díaz

**Affiliations:** 1 Department of Materno-Fetal Medicine, Genetics and Reproduction, Institute of Biomedicine of Seville (IBIS), Seville, Spain; 2 National Center of Accelerators (CNA), University of Seville, Spanish National Research Council (CSIC), Andalusian Regional Government, Seville, Spain; 3 Fetal, IVF and Reproduction Simulation Training Centre (FIRST), Seville, Spain; 4 Faculty of Medicine, University of Seville, Seville, Spain; 5 Centre for Biomedical Network Research on Rare Diseases (CIBERER), Seville, Spain

**Keywords:** advanced maternal age, assisted reproductive technologies, *in vitro* fertilization, obstetric outcomes, perinatal outcomes

## Abstract

**Objectives:**

To describe obstetric and perinatal outcomes of pregnant women with advanced maternal age (AMA) conceiving through *in vitro* fertilization (IVF) technology.

**Methods:**

A single-center prospective observational study was conducted between January and December 2024. All women aged 40 and older who conceived via IVF were included. Participants underwent regular prenatal follow-up in a maternal-fetal unit, including standardized maternal clinical assessment and fetal ultrasound evaluation. A subgroup analysis (40–44-year-old subgroup vs. ≥45-year-old subgroup) was also conducted to identify potential differences in obstetric and perinatal outcomes within our study population.

**Results:**

A total of 128 pregnant women were included. Nearly half were primigravid (*n* = 60, 46.88%) and 71 pregnancies resulted from oocyte donation (55.47%). Obstetric complications observed in this cohort included gestational diabetes mellitus (*n* = 3, 2.34%), hypertensive disorders (*n* = 6, 4.76%) preterm premature rupture of membranes (*n* = 11, 8.59%), preterm intrauterine growth restriction (*n* = 7, 5.56%) and preterm delivery (*n* = 15, 11.72%). It is noteworthy that these last three outcomes mentioned were observed significantly more frequently in the subgroup aged over 45 years. Cesarean delivery was performed in 62 cases (49.6%). Regarding neonatal outcomes, the median birth weight was 3,028 g and the median umbilical cord pH was 7.29. Eight newborns (6.45%) required admission to intensive care unit. Postnatal comorbidities were identified in 14 infants, with respiratory complications being the most frequent (12/14).

**Conclusions:**

In this cohort of women of AMA following IVF, several obstetric and neonatal complications were observed. Additionally, the subgroup analysis revealed that PPROM, preterm IUGR and preterm delivery, lower neonatal weight and respiratory complications were significantly more frequent among women aged 45 or older. These findings provide descriptive clinical data regarding pregnancies in this specific population, which remains relatively underrepresented in the literature. Given the single-center design of the study, further research, including studies with appropriate comparison groups and larger sample size conducted across multiple centers, are needed to better clarify the individual and combined contributions of AMA and IVF to these outcomes.

## 1 Introduction

Over the past three decades, the number of women postponing childbearing until their late 30s or even into their 40s has increased dramatically worldwide (1, 2). In fact, the rate of first births among women aged 35–39 increased sevenfold between 1973 and 2018, and fivefold among women aged 40–44 between 1985 and 2018 (1).

This phenomenon has been associated with a range of social, economic, and cultural factors (3, 4). Shifts in socioeconomic conditions—such as increased participation in the labor market, professional aspirations, limited workplace policies supporting motherhood and childcare, and the desire for financial independence before becoming a parent—have all been linked to this trend (5, 6).

Fertility in women begins to decline in the early 30s and decreases more rapidly after the mid to late 30s (7). Consequently, there has been a growing trend in the use of assisted reproductive technologies (ART) (8). Advances in ART -such as *in vitro* fertilization (IVF)- have increased the likelihood of successful pregnancies in older women, thereby making these procedures more common (9). This technology has effectively extended the reproductive window, leading to a higher incidence of pregnancies in women beyond the typical biological reproductive age (10, 11).

Taken together, these shifts in family structure and reproductive planning have contributed to an increased rate of pregnancies among women of advanced maternal age (AMA). Although the term AMA lacks a universally agreed-upon definition (1), it generally refers to women who are aged 35 years or older at the time of delivery, marking the later years of the reproductive lifespan (3, 5). However, because aging is a gradual and multifaceted process, establishing a precise cutoff point is challenging, especially given that age-related effects often emerge progressively (12, 13).

In fact, although women >35 years of age are traditionally associated with worse perinatal outcomes than younger women, recent studies (11, 14, 15) suggest that these findings are more evident in patients >40 years of age, highlighting the greater risk of serious maternal complications in this group.

Regardless of the cutoff used, AMA is a well-established risk factor for adverse pregnancy outcomes (14, 16). Data from population registries and large cohort studies consistently show that AMA is associated with an increased risk of maternal complications, including hypertensive disorders, gestational diabetes, placental abnormalities, and a greater need for medical intervention not only during pregnancy and delivery but also in the postpartum period—for instance, in the form of postpartum depression (7, 17). Beyond the immediate pregnancy, AMA may also negatively affect women's long-term health, as is the case of future cardiovascular disease (5).

Moreover, AMA has important implications for neonatal outcomes (1). Older maternal age is associated with higher rates of miscarriage, chromosomal abnormalities, congenital anomalies, fetal growth restriction, preterm birth, small for gestational age infants, low birthweight, and increased admissions to neonatal intensive care units (NICUs) (7, 16).

Despite the well-documented negative impacts of AMA on maternal and neonatal health, current obstetric guidelines offer limited nuance regarding the effective management of older pregnant women throughout the maternity care continuum (1). And what is more, the combined effect of AMA and IVF on obstetric and perinatal outcomes remains underexplored.

To address this gap, the present study aims to comprehensively evaluate and describe obstetric and perinatal outcomes in women aged 40 years or older who conceive through IVF.

## 2 Material and methods

### 2.1 Study design

This single-center prospective observational study was conducted at Virgen del Rocío University Hospital, a tertiary care institution and referral center for complex maternal-fetal pathology. In 2024, the hospital provided care for 9,258 patients, with a total of 4,532 births attended, 128 of them were singleton pregnancies conceived through IVF who met the inclusion criteria and were prospectively included in the study.

**Figure 1:**
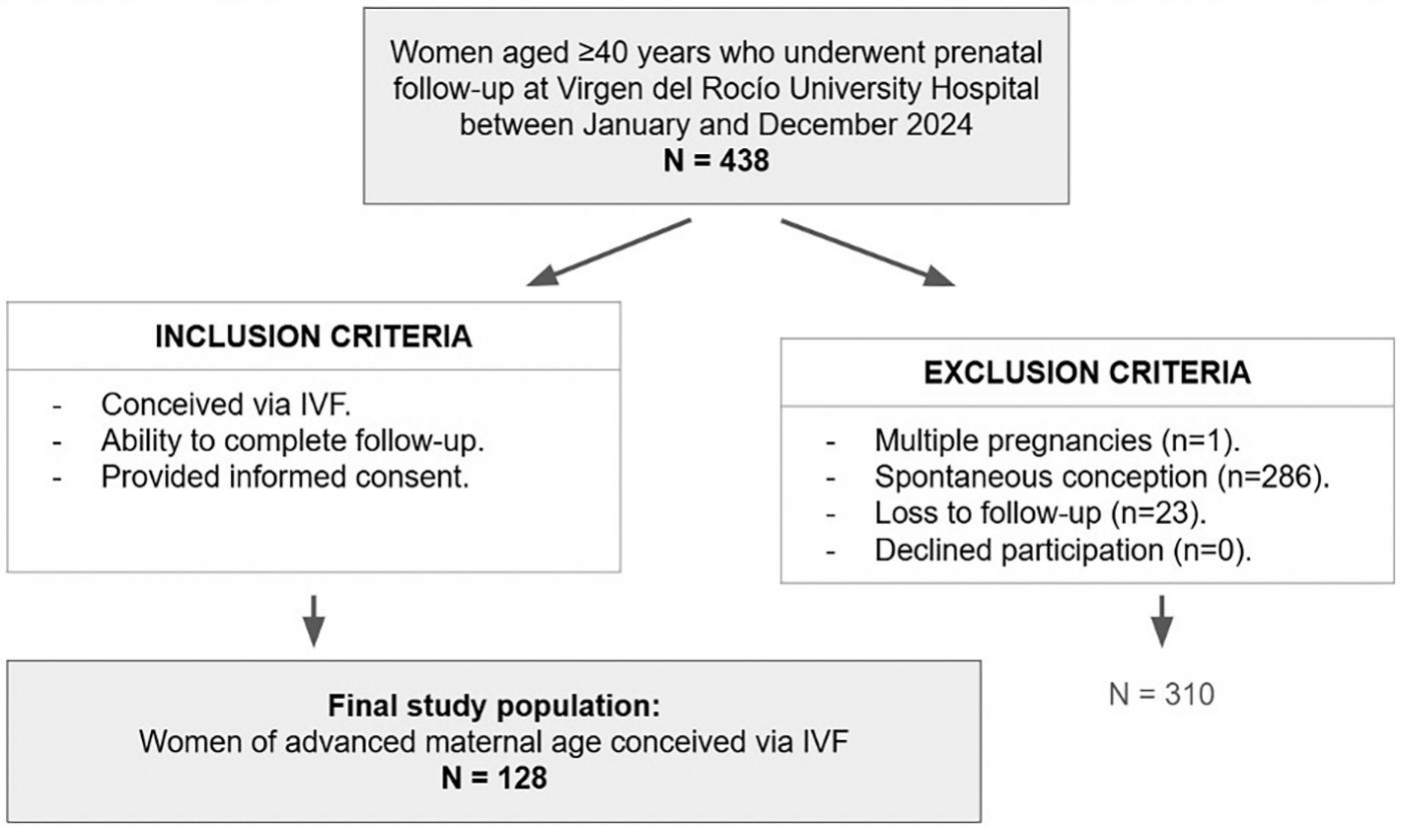
Selection process of the study.

Although advanced maternal age is traditionally defined as ≥35 years, as mentioned above, previous studies have reported a substantially increased risk of adverse obstetric outcomes among women over 40 years of age, therefore we used this age threshold for the present study (11, 15). Exclusion criteria included multiple pregnancy, inability to complete appropriate prenatal follow-up or refusal to participate. Figure 1 shows schematically the selection process and the inclusion and exclusion criteria used.

Gestational age was determined based on the date of the last menstrual period and confirmed by first-trimester ultrasound. After enrollment, participants underwent regular follow-up every four to 6 weeks by a dedicated team of maternal-fetal medicine specialists, for surveillance and early detection of obstetric complications. At each visit, maternal clinical status was assessed including blood pressure measurement and fetal development was evaluated through standardized ultrasound examinations, covering biometric parameters—Biparietal Diameter (BPD), Head Circumference (HC), Abdominal Circumference (AC) and Femur Length (FL)—and Doppler flowmetry of the uterine arteries by abdominal scan. In addition, serial blood tests including placental growth factor (PlGF), soluble fms-like tyrosine kinase-1 (sFlt-1), and the sFlt-1/PlGF ratio were performed for screening of placental dysfunction and early signs of preeclampsia.

### 2.2 Data collection

Maternal basic demographic and obstetric characteristics, delivery details, pregnancy and perinatal outcomes were collected, encompassing a total of 30 variables. These variables, along with their terminology and precise definition, are detailed in Table 1.

**Table 1:** Maternal-fetal variables included in the study.

Maternal-fetal variables	Definition	Description/value
Basic demographic characteristics		
Maternal age	Chronological age of the pregnant woman at the time of childbirth.	Discrete variable, expressed in years.
Weight	Weight of the pregnant woman at the first prenatal visit.	Continuous variable, expressed in kilograms.
Height	Height of the pregnant woman at the first prenatal visit.	Continuous variable, expressed in meters.
Body mass index (BMI)	Calculated as the woman's weight (kg) divided by the square of her height (m^2^).	Continuous variable, expressed in kilograms/metres^2^
Smoking status	Smoking during pregnancy or within three months prior to conception.	Binary variable.
Obstetric characteristics		
Gravidity	Total number of prior pregnancies at the time of assessment.	Discrete variable.
History of miscarriage	Number of previous spontaneous pregnancy losses before 23 weeks of gestation.	Discrete variable.
Parity	Number of previous deliveries at the time of the assessment.	Discrete variable.
Previous cesarean section	History of cesarean delivery in any prior pregnancy.	Discrete variable.
Previous ectopic pregnancy	History of extrauterine pregnancy prior to the current gestation.	Discrete variable.
Oocyte donation	Assisted reproductive technology where a donor provides eggs to an intended parent or couple.	Binary variable.
Obstetric complications and pregnancy outcomes		
Pregestational diabetes mellitus	Diagnosis of diabetes before woman becomes pregnant or during the first 12 weeks of gestation	Binary variable.
Gestational diabetes	Diagnosis of diabetes confirmed during pregnancy via two-step oral glucose tolerance test or abnormal glycemic profiles.	Binary variable.
Chronic hypertension	Hypertension diagnosed prior to pregnancy or before 20 weeks of gestation.	Binary variable.
Pregnancy associated hypertension	Blood pressure ≥140/90 mmHg identified after 20 weeks of gestation and resolving within 12 weeks postpartum.	Binary variable.
Preeclampsia	Hypertension accompanied by proteinuria or signs of organ dysfunction (e.g., thrombocytopenia, renal/hepatic impairment, pulmonary edema, neurological symptoms), subdivided in early (diagnosed <32 weeks of gestation) and late (diagnosed >32 weeks of gestation).	Binary variable.
Threatened preterm labor	Regular uterine contractions with cervical changes (dilatation and effacement) between 23 and 36 weeks of gestation, with intact membranes.	Binary variable.
Preterm premature rupture of membranes (PPROM)	Spontaneous rupture of membranes before the onset of labor, occurring prior to 37 weeks of gestation.	Binary variable.
Small-for-gestational age (SGA)	Birthweight below the 10th percentile but above the 3rd percentile for gestational age and sex, without Doppler abnormalities.	Binary variable.
Intrauterine growth restriction (IUGR)	Fetal growth below the 10th percentile with Doppler abnormalities or estimated fetal weight below the 3rd percentile, either early (<32 weeks) or late (≥32 weeks).	Binary variable.
Preterm delivery	Gestational age less than 37 weeks at the time of the end of the pregnancy.	Binary variable.
Placenta accreta spectrum (PAS)	Severe pregnancy complication where the placenta attaches abnormally deep into the uterine wall, directly onto the muscular layer (placenta acreta), invading the entirely myometrium (placenta increta) or even surrounding organs (placenta percreta).	Binary variable.
Placental abruption	Severe pregnancy complication where the placenta partially or completely detaches from the inner wall of the uterus before delivery.	Binary variable.
Mode of delivery	Method by which the pregnancy ended (vaginal, assisted vaginal delivery, or cesarean section).	Nominal variable.
Perinatal characteristics		
Gestational age at birth	Duration of pregnancy determined based on the date of the last menstrual period and confirmed by first-trimester scan.	Discrete variable, expressed in completed weeks + days.
Neonatal weight	Baby’s weight at birth.	Continuous variable, expressed in kilograms.
Apgar test score	Score measured at 5 min post-delivery to assess neonatal adaptation.	Discrete variable (0–10 score).
Cord blood pH	Measurement of pH in umbilical cord blood immediately after delivery.	Continuous variable.
Neonatal intensive care unit (NICU) admission	Admission of the newborn to the Neonatal Intensive Care Unit (NICU), as determined by the attending pediatrician.	Binary variable.
Neonatal morbidity	Any condition at birth potentially affecting neonatal adaptation or development.	Binary variable.

Data were extracted from electronic medical records after anonymization of sensitive information to ensure patient confidentiality. Screening for preeclampsia was performed according to the institutional protocol, based on first-trimester risk assessment and subsequent monitoring throughout pregnancy. Low-dose aspirin prophylaxis for preeclampsia prevention was prescribed following institutional and international guidelines.

### 2.3 Data processing and analysis

Data were analyzed using the Statistical Package for the Social Sciences (IBM SPSS Statistics, version 25.0). Nominal and binary variables are presented as frequencies and percentages.

Continuous variables are expressed as means with standard deviations (SD) or as medians with interquartile ranges (IQR), depending on the distribution of data. In addition, the corresponding confidence intervals for proportions and medians of the population have been calculated at a 95% confidence level. For categorical variables, the Wilson variant has been used, as it is more appropriate when proportions take on extreme values.

We also carried out a comparative analysis by dividing the study population into two subgroups based on patients' age: patients aged 40–44 years vs. those aged 45 years or older. Qualitative variables were examined using Fisher's exact test to assess possible differences between the proportions observed in both subgroups.

For quantitative variables with a normal distribution, previously assessed using the Shapiro–Wilk test, the equality of variances was evaluated using Fisher's *F*-test and, subsequently, equality of means was assessed using Student's *t*-test. In case of a non-normal distribution, the non-parametric Wilcoxon-Mann–Whitney test was applied to assess differences in the central distribution of data between the two subgroups.

### 2.4 Ethical considerations

The study was approved by the Institutional Ethics Committee of Virgen del Rocío—Virgen Macarena University Hospitals (protocol number: 1154-N-23; approval date: 22 December 2023). Written informed consent was obtained from all participants prior to enrollment and prospective follow-up. Confidentiality was strictly maintained throughout the study in accordance with the principles of the Declaration of Helsinki. The data were used exclusively for research purposes.

## 3 Results

### 3.1 Basic demographic information

Between January and December 2024, a total of 128 pregnant women were enrolled in the study. All participants received follow-up care in the Maternal-Fetal Medicine Unit of our hospital. Maternal demographic characteristics are summarized in Table 2.

**Table 2:** Maternal demographic characteristics.

Variables	Results	IC 95%
Maternal age, median (range)	42 (40–50)	(42.17, 42.91)
Weight, median (range)	65.50 (44–120)	(66.3, 71.36)
Height, median (range)	164 (153–183)	(163, 165)
BMI, median (range)	24.05 (17.5–44.9)	(24.51, 26.43)
Smoking status, *n* (%)	12 (9.38%)	(5.44, 15.67)

The median maternal age was 42 years (range: 40–50). Of the total sample, 104 women (81.25%) were aged between 40 and 45 years, while 24 women (18.75%) were aged ≥45 years. The median body mass index (BMI) was 24.05 kg/m^2^ (range: 17.5–44.9). The vast majority were non-smokers (*n* = 116, 90.62%).

### 3.2 Obstetric characteristics

Approximately half of the participants were primigravid (*n* = 60, 46.88%). Previous miscarriage was reported in 52 women (40.62%), including 19 women with two or more previous spontaneous pregnancy losses. Additionally, six women (4.68%) reported a history of ectopic pregnancy and nine had a previous cesarean section (7.03%). More than half of the participants (*n* = 71, 55.47%) conceived via ART using donated oocytes. All obstetric characteristics are exhibited in Table 3.

**Table 3:** Obstetric characteristics.

Variables	Results
Gravidity, *n* (%)	
*n* = 1	60 (46.88%)
*n* = 2 or more	68 (53.12%)
History of miscarriage, *n* (%)	52 (40.62%)
Parity, *n* (%)	
*n* = 0	112 (87.5%)
*n* = 1 or more	16 (12.5%)
Previous cesarean section, *n* (%)	9 (7.03%)
Previous ectopic pregnancy, *n* (%)	6 (4.68%)
Oocyte donation, *n* (%)	71 (55.47%)

### 3.3 Obstetric complications and pregnancy outcomes

Obstetric complications observed during pregnancy included chronic hypertension (*n* = 4, 3.12%) and hypertensive disorders of pregnancy (HDP) (*n* = 6, 4.76%), seven of which progressed to preeclampsia. There were two cases of pregestational diabetes and three cases of gestational diabetes (1.56% and 2.34%, respectively).

Preterm premature rupture of membranes (PPROM) was identified in 11 women (8.59%) and 15 pregnancies ended prematurely (11.72%). There were 11 cases of small-for-gestational age (SGA) (8.73%) and 8 cases of intrauterine growth restriction (IUGR), seven of them were early (5.56%).

Additionally, cesarean section (CS) was performed in 62 cases (49.6%), while the remaining pregnancies resulted in spontaneous vaginal delivery (*n* = 30, 24%) or assisted vaginal delivery (*n* = 32, 25.6%). A higher proportion of CS were performed on an emergency basis compared with elective procedures. Specifically, 45 emergency and 14 elective CS were recorded, while the indication could not be determined in 3 additional cases due to incomplete documentation in the patient's medical records. Among emergency CS, the most frequent indication was fetal compromise (21/45), with pathological cardiotocography (CTG) being the most common underlying reason (15/45). In contrast, elective CS were predominantly indicated due to abnormal fetal presentation, which accounted for 7 cases.

Moreover, a total of three cases of placental abruption were detected. No cases of placenta accreta spectrum were recorded. The main pregnancy outcomes with their results and confidence intervals are presented in Table 4.

**Table 4:** Pregnancy outcomes.

Variables	Results	IC 95%
Pregestational diabetes mellitus, *n* (%)	2 (1.56%)	(0.43, 5.52)
Gestational diabetes, *n* (%)	3 (2.34%)	(0.8, 6.66)
Chronic hypertension, *n* (%)	4 (3.12%)	(1.22, 7.76)
Pregnancy associated hypertension, *n* (%)	6 (4.76%)	(2.2, 10)
Preeclampsia, *n* (%)		
Early	3 (2.38%)	(0.82, 6.84)
Late	4 (3.17%)	(1.24, 7.89)
Threatened preterm labor, *n* (%)	8 (6.25%)	(3.2, 11.85)
Preterm premature rupture of membranes, *n* (%)	11 (8.59%)	(4.87, 14.73)
Small-for-gestational age, *n* (%)	11 (8.73%)	(4.94, 14.96)
Intrauterine growth restriction, *n* (%)		
Early	7 (5.56%)	(2.72, 11.02)
Late	1 (0.79%)	(0.14, 4.36)
Preterm delivery, *n* (%)	15 (11.72%)	(7.23, 18.44)
Placenta accreta spectrum, *n* (%)	0 (0%)	NA
Placental abruption, *n* (%)	3 (2.34%)	(0.8, 6.66)
Modo of delivery, *n* (%)		
Vaginal delivery	30 (24%)	(17.39, 32.19)
Assisted vaginal delivery	32 (25.6%)	(18.76, 33.9)
Cesarean section	62 (49.6%)	(41.75, 59.02)

NA, not applicable.

### 3.4 Neonatal outcomes

Regarding neonatal outcomes, the median gestational age at delivery was 39 weeks (range: 27^+4^–41^+4^ weeks), the median birth weight was 3,028 g (range: 840–4,340 g), and the median umbilical cord pH was 7.29. Eight newborns (6.45%) required admission to the NICU, with prematurity being the primary indication in all cases. Additional associated neonatal comorbidities comprised congenital malformations and surgical pathology, including one neonate with Tetralogy of Fallot, duodenal atresia, and esophageal atresia. Neonatal comorbidities were identified in 14 infants, with respiratory complications being the most common, accounting for 12 of these cases (6 cases of respiratory distress syndrome, 5 cases of transient tachypnea of the newborn and 1 case of neonatal bronchiolitis). There were two cases of neonatal sepsis. Neonatal outcomes are detailed in Table 5.

**Table 5:** Neonatal outcomes.

Variables	Results	IC 95%
Gestational age at birth, median (range)	39 (27^+4^–41^+4^)	(38–38^+6^)
Neonatal weight, median (range)	3,028 (840–4,340)	(2,909, 3,146)
Apgar test score, median (range)	9 (5–10)	NA
Cord blood pH, median (range)	7.29 (7.09–7.4)	(7.28, 7.31)
ICU admission, *n* (%)	8 (6.45%)	(3.31, 12.22)
Neonatal morbidity, *n* (%)	14 (11.29%)	
Respiratory complications	12 (9.68%)	(5.61, 16.16)
Septicemia	2 (1.61%)	(0.44, 5.69)

NA, not applicable.

### 3.5 A closer approach to women age: 40–44-years-old subgroup vs. ≥45 years-old subgroup

We carried out a subgroup analysis to identify potential differences in obstetric and perinatal outcomes within our study population. For that purpose, our population was divided into two subgroups: patients aged between 40 and 44 years (*n* = 104, 81.25%), and patients aged 45 years or older (*n* = 24, 18.75%).

The subgroup analysis suggested that in the older subgroup (≥45 years), there was a significantly higher rate of PPROM (*p*-value 0.006), preterm IUGR (*p*-value 0.024) and preterm delivery (PTD) (*p*-value 0.036).

In addition, neonatal respiratory complications appeared to be higher (*p*-value 0.046), whilst neonatal weight at birth was lower (*p*-value 0.024) among women aged 45 or older. No other variable examined showed any significant difference, with the exception of height (higher in the 40–44-year-old group) and donor status (higher in the ≥45-year-old group).

The results of the subgroup analysis are presented in Table 6.

**Table 6:** Subgroup analysis.

Variables	Subgroup 40–44 years (104) (81.25%)	Subgroup ≥45 (24) (18.75%)	*p*-value
Maternal demographic characteristics			
Maternal age, median (range)	42 (40–44)	45.5 (45–50)	<0.001
Weight, median (range)	67 (44–115)	60.5 (46–120)	0.146
Height, median (range)	166 (153–183)	160 (153–172)	**0.005**
BMI, median (range)	24.45 (17.5–44.9)	23.15 (17.7–42.5)	0.746
Smoking status, *n* (%)	10 (9.62%)	2 (8.33%)	1.00
History of miscarriage, (%)	42 (40.38%)	10 (41.67%)	1.00
Previous cesarean section, *n* (%)	5 (4.81%)	4 (16.67%)	0.062
Previous ectopic pregnancy, *n* (%)	2 (1.92%)	2 (8.33%)	0.188
Oocyte donation, *n* (%)	53 (50.96%)	18 (75%)	**0.04**
Pregnancy outcomes			
Pregestational diabetes mellitus, *n* (%)	1 (0.96%)	1 (4.17%)	0.341
Gestational diabetes, *n* (%)	2 (1.92%)	1 (4.17%)	0.466
Chronic hypertension, *n* (%)	3 (2.88%)	1 (4.17%)	0.569
Pregnancy associated hypertension, *n* (%)	6 (5.88%)	0 (0%)	0.594
Preeclampsia, *n* (%)			
Early	1 (0.96%)	2 (8.33%)	0.096
Late	4 (3.85%)	0 (0%)	1.00
Threatened preterm labor, *n* (%)	5 (4.81%)	3 (12.5%)	0.17
Preterm premature rupture of membranes, *n* (%)	5 (4.81%)	6 (25%)	**0.006**
Small-for-gestational age, *n* (%)	10 (9.8%)	1 (4.17%)	0.689
Intrauterine growth restriction, *n* (%)			
Early	3 (2.94%)	4 (16.67%)	**0.024**
Late	1 (0.98%)	0 (0%)	1.00
Preterm delivery, *n* (%)	9 (8.65%)	6 (25%)	**0.036**
Placenta accreta spectrum, *n* (%)	0 (0%)	0 (0%)	NA
Placental abruption, *n* (%)	3 (2.88%)	0 (0%)	1.00
Mode of delivery, *n* (%)			
Vaginal delivery	27 (26.47%)	5 (21.74%)	0.794
Assisted vaginal delivery	27 (26.47%)	3 (13.04%)	0.279
C-Section	48 (47.06%)	15 (65.22%)	0.166
Neonatal outcomes			
Gestational age at birth, median (range)	38^+3^ (38–39)	36^+6^ (35^+4^–38^+1^)	**0.001**
Neonatal weight, median (range)	3,180 (1,228–4,340)	2,990 (840–4,105)	**0.024**
Cord blood pH, median (range)	7.29 (7.09–7.39)	7.3 (7.16–7.4)	0.311
ICU admission, *n* (%)	5 (4.95%)	3 (13.04%)	0.166
Neonatal morbidity, *n* (%)			
Respiratory complications	7 (6.93%)	5 (21.74%)	**0.046**
Septicemia	1 (0.99%)	1 (4.35%)	0.338

The values highlighted in bold correspond to statistically significant results (p<0.005).

## 4 Discussion

This study describes obstetric and neonatal outcomes in a cohort of women aged 40 years or older who conceived through IVF and were followed in a tertiary maternal–fetal medicine unit. Several obstetric and neonatal complications were observed in this population during pregnancy and delivery.

Our results showed obstetric complications including gestational diabetes, HDP, PTD, and CS, consistent with outcomes previously reported in women of AMA undergoing IVF (9, 15).

AMA is a well-established independent risk factor for first-trimester miscarriage, even after controlling for parity or a history of previous pregnancy loss (16). The rate of first-trimester pregnancy loss ranges from 17% to 25% in women aged 35–40, increases to 33%–51% in those aged 40–45, and reaches 57%–75% in women over 45 years (6, 16). The proportion of women with a history of previous miscarriage in our study (40.62%) is consistent with these previously reported rates. Previous studies have shown that most pregnancy losses in AMA occur between 6 and 14 weeks of gestation (5). These events are frequently associated with chromosomal abnormalities such as trisomies and other aneuploidies, which have been linked to age-related declines in oocyte quality and meiotic competence (5).

Regarding diabetes, previous studies have reported that the risk of both pre-existing and gestational diabetes is three to six times higher in women over 40 years of age compared to those aged 20–29 (5). In the general obstetric population, the incidence of gestational diabetes is approximately 3%, whereas it rises to 7%–12% in women over 40 and can reach up to 20% in those over 50 (5).

In our cohort, gestational diabetes was observed in 2.34% of pregnancies. However, a closer look to our women age subgroups (40–44-year-old vs. ≥45-year-old) revealed that the rate of pregestational, and gestational diabetes rose from 0.96% and 1.92%, respectively, in the 40–44-year-old subgroup to 4.17% in the ≥45-year-old subgroup, but no statically significant differences were obtained (*p*-value 0.341 and 0.466, for each comparison).

Data from general obstetric population estimate the incidence of preeclampsia at 3%–4%, rising to 5%–10% among women aged over 40, and up to 35% in those aged over 50 (5, 18). Similar findings were observed in our cohort, where 8.33% of pregnancies among women aged 45 or older were complicated by early preeclampsia. This association is supported by both an earlier systematic review and a recent meta-analysis focusing on women over the age of 40, which demonstrated a 1.5–2.0 times higher risk of developing preeclampsia in this population compared to their younger counterparts, regardless of parity (16, 19).

About CS rate, previous studies have reported a linear relationship between AMA and the likelihood of cesarean delivery, without a clear threshold effect (16). This pattern has been consistently observed across different healthcare settings, although the magnitude of the association may vary depending on the population studied (16).

A previous study reported progressively higher CS rates with AMA, particularly among women aged over 40 years, with a further increase observed at older maternal ages (5). In our cohort, a similar trend was observed, with cesarean delivery being more frequent in the ≥45-year-old subgroup (65.22%) compared with the 40–44-year-old subgroup (47.06%), although this difference did not reach statistical significance (*p*-value 0.166).

Previous studies evaluating ART pregnancies have also described elevated CS rates, particularly among older and primiparous women (20–23). Nevertheless, further comparative studies are needed to better evaluate obstetric outcomes in IVF-treated pregnancies, particularly regarding cesarean delivery and the factors that may contribute to the choice of delivery mode (24).

Regarding PTD, we observed a rate of 11.72%, which was higher than that reported in the general obstetric population (4.1%) (13). It is noteworthy, that PTD was more frequent among women aged 40–44 years (*n* = 9, 8.65%) compared to those ≥45-year-old (*n* = 6, 25%). The differences between the study subgroups were significant (*p*-value 0.036).

In line with the observed rates of preterm birth and other obstetric complications, 6.45% of newborns in our cohort required admission to NICU, ranging from 4.95% among women aged 40–44 to 13.04% in women aged 45 and over, without reaching significant differences (*p*-value 0.166). These findings are consistent with previous studies, that have reported increasing NICU admission rates with advancing maternal age, with rates of approximately 6% among women aged 45–46 years (25). Similarly, Pinheiro et al. reported a higher risk of NICU admission among neonates born to AMA mothers aged >40 years (OR 1.20; 95% CI: 1.13–1.27; *I*^2^ = 0%) compared to those born to women aged 35–40 years (OR 1.13; 95% CI: 1.09–1.18; *I*^2^ = 47%) (26).

Moreover, a study by Newman et al. examining the effects of IVF in women aged 45 years or older found that neonates conceived through IVF were significantly more likely to require NICU admission and had a longer length of hospital stay (>4 days) (27).

In this sense, IVF has been independently associated with adverse obstetric and perinatal outcomes, including HDP and PTD (8). IVF pregnancies have also been linked to congenital anomalies, imprinting disorders, and neurodevelopmental conditions, as well as to adverse health outcomes that may persist beyond the neonatal period, such as obesity, hypertension, and diabetes (8). Further comparative studies are needed to better evaluate the association between IVF pregnancies in women of AMA and adverse neonatal outcomes such as those mentioned above.

Finally, a more in-depth analysis of IVF-related results, focused on embryological outcomes, revealed that different hormonal stimulation protocol, could lead to differences in embryo quality. Thi et al. (28) found a significant increase in the number of day 3 and day 5–6 embryos and good-quality blastocysts from follitropin delta plus clomiphene citrate cycles compared to follitropin alpha cycles. Although improved embryo quality represents a beneficial aspect, it remained uncertain if these improvements could be translated into improved pregnancy outcomes.

Our findings suggest that pregnancies in women of AMA conceived through IVF represent a clinically complex population. As the number of pregnancies at AMA and the use of ART continue to increase worldwide, further research will be needed to better characterize the clinical course of these pregnancies and to identify strategies that may help improve perinatal care.

### Strengths and limitations

Among the strengths of this study is the prospective description of a cohort of women aged ≥40 years who conceived through IVF, a population that remains relatively underrepresented in the literature despite its increasing prevalence. This study also provides clinically relevant information regarding obstetric and neonatal outcomes in this specific subgroup.

Furthermore, the prospective data collection may have contributed to improved consistency and completeness of the recorded clinical information while reducing potential recall bias. In addition, the study was also strengthened by a clear inclusion criteria and the use of a standardized maternal care protocol consistently applied throughout the study period.

Important limitations should be also considered when interpreting our results. First, the single-center design may limit external generalizability of our findings, as the characteristics of AMA women in our institution may differ from those in other healthcare environments or geographic regions. Additionally, the absence of a control group of women of the same age with natural conception or younger women undergoing IVF reduces the ability to conduct robust comparative analyses, distinguish the independent effects of AMA and IVF from their interaction or their potential synergistic effect. The relatively small sample size, along with the limited study period and single-center scope, may reduce the statistical power of subgroup analyses.

Moreover, although multiple clinically relevant variables were collected, the possibility of residual confounding cannot be excluded. Factors such as maternal comorbidities, lifestyle characteristics, or IVF-related parameters including embryo transfer characteristics, infertility etiology, and number of IVF cycles were not systematically available and therefore could not be fully evaluated in the present analysis. Finally, although prospective data collection was performed, some variables contained missing data due to incomplete documentation in routine clinical records.

## 5 Conclusions

In this prospective cohort of women aged ≥40 years who conceived through IVF, several obstetric and neonatal complications were observed, including gestational diabetes, HDP, PTD, CS, and NICU admission, among others. Additionally, the subgroup analysis revealed that PPROM, preterm IUGR and PTD, LWB and respiratory complications were significantly more frequent among women aged 45 or older.

These findings provide descriptive clinical data regarding pregnancies in this specific population, which remains relatively underrepresented in the literature.

Given the single-center design of the study, further research, including studies with appropriate comparison groups and larger sample size conducted across multiple centers, are needed to better clarify the individual and combined contributions of AMA and IVF to these outcomes.

## Data Availability

The raw data supporting the conclusions of this article will be made available by the authors, without undue reservation.
